# Breeding Buckwheat for Increased Levels of Rutin, Quercetin and Other Bioactive Compounds with Potential Antiviral Effects

**DOI:** 10.3390/plants9121638

**Published:** 2020-11-24

**Authors:** Zlata Luthar, Mateja Germ, Matevž Likar, Aleksandra Golob, Katarina Vogel-Mikuš, Paula Pongrac, Anita Kušar, Igor Pravst, Ivan Kreft

**Affiliations:** 1 Biotechnical Faculty, University of Ljubljana, Jamnikarjeva 101, SI-1000 Ljubljana, Slovenia; zlata.luthar@bf.uni-lj.si (Z.L.); mateja.germ@bf.uni-lj.si (M.G.); matevz.likar@bf.uni-lj.si (M.L.); aleksandra.golob@bf.uni-lj.si (A.G.); katarina.vogelmikus@bf.uni-lj.si (K.V.-M.); paula.pongrac@gmail.com (P.P.); 2 Jožef Stefan Institute, Jamova 39, SI-1000 Ljubljana, Slovenia; 3 Nutrition Institute, Tržaška 40, SI-1000 Ljubljana, Slovenia; anita.kusar@nutris.org (A.K.); igor.pravst@nutris.org (I.P.)

**Keywords:** breeding, buckwheat, flavonoids, rutin, quercetin, emodin, fagopyrin, antiviral activity

## Abstract

Common buckwheat (*Fagopyrum esculentum* Moench) and Tartary buckwheat (*Fagopyrum tataricum* (L.) Gaertn.) are sources of many bioactive compounds, such as rutin, quercetin, emodin, fagopyrin and other (poly)phenolics. In damaged or milled grain under wet conditions, most of the rutin in common and Tartary buckwheat is degraded to quercetin by rutin-degrading enzymes (e.g., rutinosidase). From Tartary buckwheat varieties with low rutinosidase activity it is possible to prepare foods with high levels of rutin, with the preserved initial levels in the grain. The quercetin from rutin degradation in Tartary buckwheat grain is responsible in part for inhibition of α-glucosidase in the intestine, which helps to maintain normal glucose levels in the blood. Rutin and emodin have the potential for antiviral effects. Grain embryos are rich in rutin, so breeding buckwheat with the aim of producing larger embryos may be a promising strategy to increase the levels of rutin in common and Tartary buckwheat grain, and hence to improve its nutritional value.

## 1. Introduction

Common buckwheat (*Fagopyrum esculentum* Moench) and Tartary buckwheat (*Fagopyrum tataricum* (L.) Gaertn.) are traditionally grown in the Himalayas, south-western and northern China, Korea, Japan, and central and eastern Europe [1,2]. Two types of buckwheat are mainly used around the world: common buckwheat and Tartary buckwheat. Which buckwheat species is used depends on the production zone and the way of utilization. Generally, in Europe, the USA, Canada, Brazil, South Africa and Australia, the common buckwheat prevails. This holds true for most Asian buckwheat-growing countries, e.g., Japan, Korea, and the central and northern parts of China. Tartary buckwheat is grown and used in the mountain regions of Himalaya. In northern India, Bhutan and Nepal both types are known, yet Tartary buckwheat is grown in harsher climatic conditions. Tartary buckwheat flour is recently increasingly used in preparing dishes due to its even higher content of rutin in comparison to of common buckwheat. 

Buckwheat is a low-input plant, suitable for growing in harsh ecological conditions, decreasing the density of invasive wireworms, with ecotypes showing preharvest sprouting resistance, shattering resistance, and lodging resistance [3,4]. Buckwheat is expected to be an even more important plant in agriculture and a dish in the cuisine and dietary habits in many countries, including those of Asia, Europe and America [5]. Besides the agricultural, cultural and culinary value of buckwheat, recently more emphasis has been placed on its health-related and nutritional value; well-balanced amino acid composition of proteins, dietary fiber, content of mineral elements and vitamins, as well as rich content of diverse antioxidants, mainly flavonoids rutin and quercetin [6,7]. Novel buckwheat dishes and products, including diverse pasta products, grain-based products, sprouts and young plants, have potential in Asia and in Europe.

Common buckwheat and Tartary buckwheat are used in different parts of the world to make various food products. One of reasons for buckwheat’s popularity is the ability to repress weeds and the relative resistance of buckwheat to pests and diseases [3,8]. Buckwheat is thus suitable for biological (organic, ecological) cultivation. Buckwheat groats are a prebiotic food because they have increased amounts of resistant starch [9].

Buckwheat does not contain gluten proteins, so it is safe for people who require a gluten-free diet [10]. Buckwheat can increase food diversity, evokes tradition and in a way revives the heritage of “the good old days”. 

While common buckwheat breeding has been widespread in Japan, China, Korea, Poland, Ukraine, Belarus, Russia and Slovenia, new Tartary buckwheat varieties have been released only recently in China, Japan and Slovenia. Until now, the breeding of common and Tartary buckwheat has focused on achieving high yields and resistance to unfavourable environmental conditions. As these buckwheat species are both good sources of polyphenolic compounds, more attention is now being directed towards optimisation of their nutritional quality parameters, and increasing their content of flavonoids (e.g., rutin, quercetin) and other phenolics [11]. In this review, we will appraise recent attempts to develop common and Tartary buckwheat for grain with higher flavonoid and emodin levels.

## 2. Rutin, Rutinosidase and Quercetin

Flavonoids and their glycosides represent one of the major classes of plant secondary metabolites. These are widely distributed natural compounds of special interest, due to their antioxidant properties and their potential in the prevention of tiredness, diabetes mellitus, oxidative stress and Parkinson’s disease [6,12,13,14,15]. Flavonoids are a group of plant-derived phenolic compounds, with a 15-carbon skeleton, made up of two benzene rings and bound to a heterocyclic pyrone ring. Rutin (i.e., 3′,4′,5,7-tetrahydroxy-flavone-3-rutinoside) is a flavonol glycoside that has received much attention due to its antimicrobial, anti-inflammatory, anticancer and antidiabetic properties [6,16,17], while quercetin is an aglycone formed after enzymatic degradation of rutin by rutinosidase. Orally admistered quercetin is able to cross the blood–brain barrier and accumulate in brain tissue [17]. 

**Figure 1:**
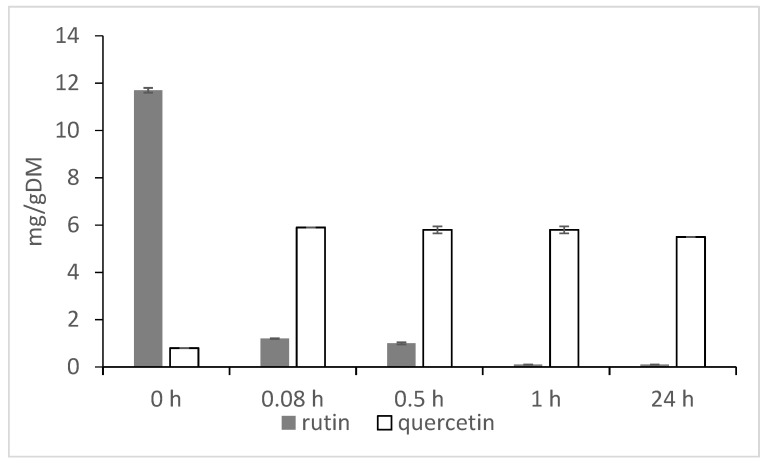
Rutin transformation to quercetin in buckwheat flour under condition of 44% water in the mixture [25].

One of the functions of rutin in buckwheat plants is protection of the plant organs from solar ultraviolet (UV) radiation [18,19]. In addition, quercetin derivatives were detected among the main bioactive substances in buckwheat root exudates, protecting buckwheat plants from weeds [20,21]. Exposure of buckwheat grain to moisture results in enzymatic breakdown of rutin to quercetin by rutinosidase (Figure 1), as happens also after milling and mixing of the flour with water [19,22,23,24,25]. Vombergar [25] simulated the procedure of dough and bread making from Tartary buckwheat flour, by mixing 66% of flour and 44% of water. The mixture was covered to maintain the moisture and was stored at 20 ºC for 0.08, 0.5, 1, respectively, for 24 hours. After the given time of storage the concentration of rutin and quercetin was measured by high-performance liquid chromatography (HPLC). Within first 5 min (0.08 h) most of rutin was degraded, and quercetin appeared in the flour/water mixture. The concentration of quercetin was after that stable for at least 24 hours (Figure 1).

Common and Tartary buckwheat grain and leaves are important sources of rutin [6,26,27,28,29,30] and food products like bread, groats, noodles and pancakes [11]. In humans, products derived from common and Tartary buckwheat grain have been shown to reduce fatigue symptoms, decrease blood cholesterol levels, and improve lung capacity [12,13,31,32,33]. Buckwheat grain methanol extracts can also protect DNA from damage caused by hydroxyl radicals, as was demonstrated for a human hepatoma cell line [34]. These DNA-protecting effects of buckwheat extracts have been associated with high levels of rutin and quercetin, although other substances in buckwheat extracts might also have roles [6,34]. Rutin-derived quercetin is also a stronger inhibitor of aflatoxin synthesis in grain by *Aspergillus flavus*, compared to rutin [35].

A new Tartary buckwheat variety with lower rutinosidase activity was recently bred in Japan [36,37,38,39,40]. The motivation for this was to produce Tartary-buckwheat-based food products (e.g., bread, noodles) that contain considerably more rutin than quercetin, in order to achieve more acceptable foods (i.e., less bitter) from Tartary buckwheat [41,42,43,44,45]. Furthermore, the consumption of rutin-rich foods offers a feasible approach for the improvement of human nutrition, due to the known protective health effects of rutin. This has now also become especially important because of its antiviral activity, including its reported activity against the Sars-CoV-2 virus [46,47]. Buckwheat is a promising source of rutin, the inhibitor of main protease and other protein targets of Sars-CoV-2 virus [48,49,50].

Tartary buckwheat with low rutinosidase activity may become more popular in countries where foods made from Tartary buckwheat are generally less popular than those made from common buckwheat. This can occur because the less-bitter buckwheat products are preferred by consumers, which is especially the case in Japan, where consumers are used to non-bitter buckwheat noodles. For markets where consumers prefer the ‘gentler’ taste of common and Tartary buckwheat dishes, the backcrossing of Tartary buckwheat varieties with the new low-rutinosidase Tartary buckwheat variety would represent an important tool to provide foods with high content of flavonoids, but without a bitter taste. 

**Figure 2:**
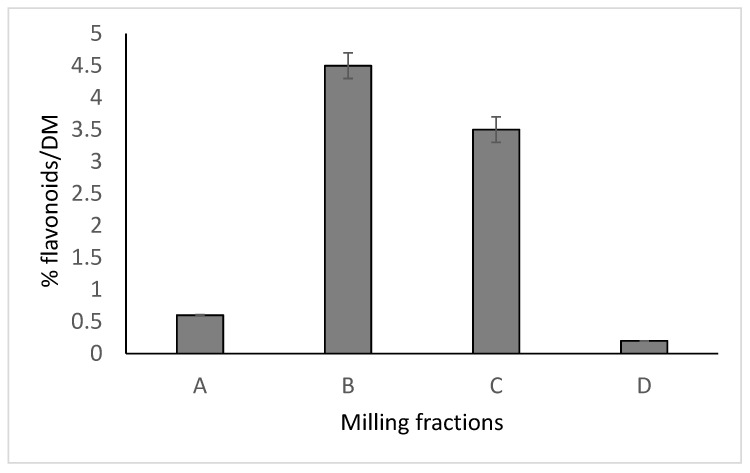
Distribution of flavonoids among Tartary buckwheat milling fractions.

Another way to prevent degradation of rutin to quercetin is to scald Tartary buckwheat flour with water and keep it wet for 20 min at the temperature 80–95 °C [51]. This process helps to conserve more of the rutin in the flour, which results in higher levels of rutin in the final product, such as bread or noodles [51,52,53]. In addition, a lot of the rutin in common and Tartary buckwheat is stored in the embryo. Therefore, breeding of buckwheat with the aim of producing larger embryos may be a promising strategy for increasing the levels of rutin in common and Tartary buckwheat grain (Figure 2, Table 1) [11]. 

Rutin and quercetin are of similar importance from the point of view of impact to human body [54,55]. However, the enhancement of rutin content in buckwheat grain is of great importance to avoid the bitter taste of quercetin [41,42,43].

**Table 1:** Rutin and quercetin content in common and Tartary buckwheat grain and products (in g/100g dry matter).

Species	Sample	Rutin	Quercetin	Literature Source
Tartary buckwheat	Flour	1.46	0.19	[52]
Tartary buckwheat	Bread		0.51	[52]
Tartary buckwheat	Herb	1.2–3.1		[56]
Tartary buckwheat	Grain	1.3–1.6		[56]
Common buckwheat	Grain	0.01		[56]
Common buckwheat	Flour	0.0003		[23]
Tartary buckwheat	Wholemeal flour	0.22	0.19	[57]
Tartary buckwheat	Malt from wholemeal flour	0.37	0.41	[57]
Tartary buckwheat	Flour	1.17	0.06	[25]
Tartary buckwheat	Flour	1.584–1.637		[42]
Common buckwheat	Grain	0.017-0.070		[58,59]

Vombergar et al. [60] performed milling, sieving and analysing experiments with Tartary buckwheat grain (Figure 2). Among milling fractions, the highest concentration of flavonoids in Tartary buckwheat flour (granulation over 100 µm up to including 1000 µm) was established as 3.5–4.5% flavonoids in dry matter. This was a milling fraction of dark coarse flour, containing parts of milled grain embryo. Knowledge about the distribution of flavonoids among milling fractions, in relation to the size of the particles, is important in obtaining flavonoid-rich milling fractions of high nutritional relevance [60]. 

A, flour, granulation ≤100 μm; B, flour, granulation 100 μm < x ≤ 236 μm, including embryo particles; C, flour, granulation 236 μm < x ≤ 1000 μm; D, flour, granulation >1000 μm, including bran and husk [60].

The novel Tartary buckwheat variety with low rutinosidase activity also shows no α-glucosidase inhibition, while the traditional Tartary buckwheat varieties are characterised by higher levels of α-glucosidase inhibitory activity [36,61]. Indeed, in Tartary buckwheat grain, quercetin originating from rutin degradation has been reported to be responsible for α-glucosidase inhibitory activity [14]. This is important for mitigation of the diabetes mellitus condition [14,61,62,63]. High rutin and low quercetin buckwheat products are important only for some customers, for example consumers of soba (buckwheat) noodles in Japan. On other hand, European customers, accepting some bitter dishes, may have the benefit of mitigation of the diabetes mellitus condition by the α-glucosidase inhibitory activity of quercetin [14]. 

## 3. Fagopyrin and Emodin

Fagopyrin is another secondary metabolite that is found in the green plant parts (e.g., sprouts, leaves) and less so in the grain [64,65,66]. Six fagopyrin derivatives were identified in three species of *Fagopyrum* (i.e., *F. esculentum*, *F. tataricum*, *F. cymosum*), as fagopyrins A to F [10,67]. The highest fagopyrin levels have been reported for *F. cymosum* flowers, at 20.7 mg/g dry weight, with high levels also found in common buckwheat flowers and leaves, at ≤4.83 mg/g and 0.32 to 2.3 mg/g, respectively [66]. 

The levels of fagopyrin are at their highest during seed germination, and light is important for the transformation of protofagopyrins to fagopyrins, as increased fagopyrin levels have been shown to accompany increased light conditions [10,67,68]. Fagopyrin is involved in the regulation of the mycelial growth, morphology and pathogenicity of fungi [69]. The consumption of large quantities of the green parts of buckwheat plants can also provoke fagopyrism, a condition of photosensitisation that can cause skin irritation, oedema and a serous exudate. In buckwheat grain, however, fagopyrin levels are lower than those of other antioxidative compounds, thus avoiding these negative effects on human health [10]. 

The potential to reduce fagopyrin levels in buckwheat by plant breeding has not been assessed to date. However, as fagopyrin appears to have a role in the protection of plants against UV radiation, pests and/or diseases, breeding for varieties with reduced fagopyrin levels might result in collateral, undesired, effects for plants grown in high UV environments.

Emodin (i.e., 6-methyl-1,3,8-trihydroxyanthraquinone) is another buckwheat metabolite that has structural similarity to metabolites from the group of naphthodianthrones. It is believed to be a precursor of hypericin [70], and possibly also the precursor of fagopyrin. Emodin isolated from Tartary buckwheat grain [71] has been shown to bind well to all three active sites of the RNA binding domain of the nucleocapsid phosphoprotein of Sars-CoV-2 [70,72,73]. However, the biosynthesis pathways of emodin in Tartary buckwheat remain unclear to date [74]. Emodin has been detected in Tartary buckwheat bran and leaves, but not in the roots [71]. Molecular modelling has suggested that hypericin can interact with HIV-1 protease [75], although further studies of the antiviral effects of fagopyrin and/or buckwheat metabolites resembling hypericin still need to be performed.

## 4. Buckwheat Breeding: Challenges and Prospects for the Future

The breeding methods for common and Tartary buckwheat differ considerably. Tartary buckwheat is self-compatible, while common buckwheat is an obligatory cross-fertilising plant. Large numbers of the original domestic buckwheat varieties can still be found in countries with a long tradition of buckwheat cultivation and consumption. In particular, wild relatives of common and Tartary buckwheat can be found in the Himalayas, where the *Fagopyrum* genus originated. 

To breed buckwheat varieties with agronomically and nutritionally improved characteristics, it is important to collect as much of the available genetic material as possible, and to store it appropriately. Due to the wide phenotypic and genetic heterogeneity of common buckwheat [76,77], the search for valuable but recessive genes in this heterozygous plant will be challenging, but will also be worth the effort both scientifically and commercially. In common buckwheat, it is possible to reveal properties under the control of recessive genes in the progeny after crossing sister plants. 

Breeding of common buckwheat is difficult because of a single gene complex S-locus, which controls self-incompatibility, as it is tied to favorable weather conditions during flowering, which influence the presence of pollinators [78,79,80]. An optimally designed breeding program includes an appropriate initial pin/thrum ratio of parent plants in favor of thrum plants. In buckwheat, nectar production can be influenced by heteromorphy, ploidy level, plant age, inflorescence position and abiotic factors. Except for the morphology of the reproductive organs, both morphs differ in nectar production. Thrum flowers or plants produce up to 30% more nectar than pin flowers during the first half part of the flowering [81]. Pollination efficiency depends on many factors: a plant’s capacity to attract pollinators by flower morphology, and by pollen and nectar production on the insect (honeybee) abundance and ability to collect, transport, and deposit pollen on a compatible stigma. Apart from pollinator-related pollination, common buckwheat is very sensitive to climatic factors, sowing date, photoperiod sensitivity and local agronomic practices that have a strong impact on yield of seeds. Abiotic factors like weather conditions, drought, solar radiation, weeds and available nutrients may affect the development and yield of buckwheat plants [81,82,83]. Some of the important breeding objectives in common buckwheat breeding include stable yield, superior seed quality, lodging resistance, determinate growth habit, easy dehulling, low shattering of seeds, flood resistance, rutin content, low allergenic protein content, good aroma, and pre-harvest sprouting resistance [2,4,5,58,59,84,85]. 

In contrast to common buckwheat, Tartary buckwheat is homozygous, which essentially means that Tartary buckwheat populations are more conserved and that there are no ‘hidden genes’ in the population. Indeed, being homozygous, Tartary buckwheat populations consist of plants that produce genetically identical progeny. In domestic populations of Tartary buckwheat, there are inbred lines that can differ in terms of specific characteristics, including the levels of desired metabolites and also the crop yield, easy dehulling, low shattering seeds [86]. Thus, the metabolite levels are not the sole criterion for selection. Both the grain yield per unit area and the yield of metabolites per unit area are of paramount importance. Certainly, for extraction and isolation of a metabolite in the pharmaceutical industry (e.g., rutin), it is important to reach high plant levels of the metabolite, to minimise the harvested plant material needed, and to optimise the extraction, isolation and concentration of the desired metabolite [5,87,88].

In chemical analyses of mixtures of Tartary buckwheat seeds, it is not easy to find genotypes with specific desired properties and an appropriate metabolite layout in such bulk material. What is needed is the analysis of seeds from individual plants, or from genetically identical sister plants. Otherwise any outstanding (and potentially desired) properties of individual outliers would be masked by the impact of the metabolite concentrations of the plants of average quality [84,89]. 

Tartary buckwheat can by ploidy induction be effectively transformed to the tetraploid level by colchicine or other cytostatics. In comparison to the diploid plants, various agronomic traits may be increased in the induced tetraploid plants, including seed size, 1000-seed mass, leaf size, chlorophyll content, flower size and pollen diameter. The content of seed protein and flavonoid may also be increased in the tetraploid plants. It is very important to choose a genotype with a high yield and easily shelled traits before polyploidization [90,91]. Perennial buckwheat development could be an interesting challenge in buckwheat breeding [87].

**Figure 3:**
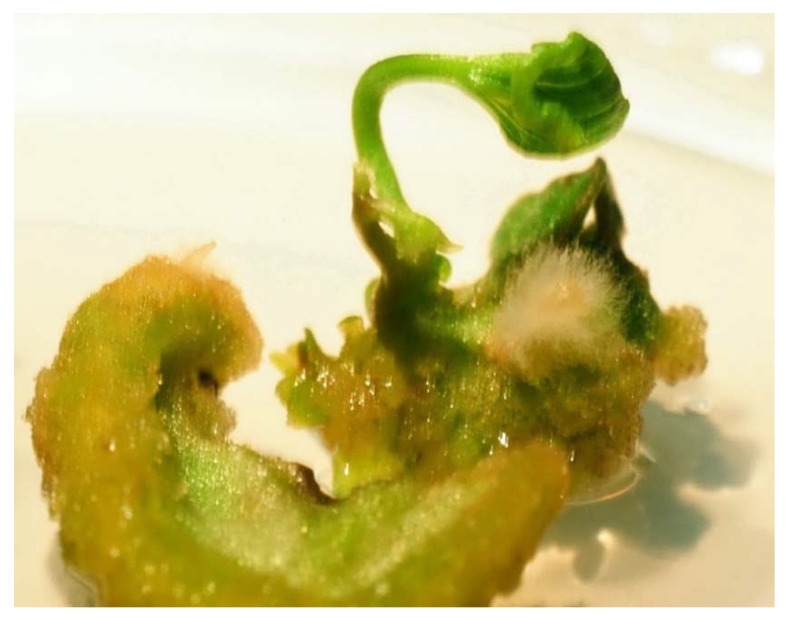
Common buckwheat cotyledons can be used in tissue culture techniques to regenerate several progeny plants with an identical genotype.

Tissue culture techniques can be applied to the breeding of common and Tartary buckwheat. Using in vitro tissue culture, whole genetically identical plants can be regenerated from a single cotyledon [92,93,94,95], a hypocotyl [96,97] or a meristem [98]. In this way, haploids of common buckwheat have already been generated [99,100]. However, the success rate of regeneration is low and greatly depends on the genotype [94]. Therefore, it will be necessary to develop rapid and easy procedures with high regeneration rates and reproducibility. Interspecies hybrids can also be successfully obtained by rescuing immature embryos [101,102]. Such haploids would allow the breeding of hybrids, although the procedures for obtaining haploids in buckwheat have not yet been optimised. Furthermore, the regeneration of buckwheat plants from tissue cultures is limited by the high levels of phenolic substances in common and Tartary buckwheat tissues (Figure 3).

However, interspecies hybrids within the genus *Fagopyrum* and their resulting alloploids [103] represent good sources for breeding, to obtain plants with large grain embryos and promising flavonoid and emodin levels.

## 5. Conclusions

Common buckwheat and Tartary buckwheat grain contain the bioactive compounds rutin, quercetin, emodin and fagopyrin with potential antiviral effects. Rutin, a flavonol glycoside is important because of its reported antimicrobial, anti-inflammatory and anticancer properties. Quercetin is an aglycone formed after enzymatic degradation of rutin by rutinosidase. In addition to the properties of rutin, it has been reported that the antidiabetic effects of quercetin are related to its α-glucosidase inhibitory activity. This is important for the mitigation of diabetes mellitus. In Tartary buckwheat grain, quercetin originating from rutin degradation is responsible for the bitter taste of products and foods. The prevention of rutin degradation to quercetin is important for customers, especially in Asian countries, who are not familiar with the bitter taste of buckwheat dishes. In contrast, customers who accept bitter dishes, can benefit from the mitigation of a diabetes mellitus condition by the α-glucosidase inhibiting effect of quercetin. 

Fagopyrin, a naphthodianthrone, is involved in the regulation of mycelial growth, the morphology and pathogenicity of fungi, and in the protection of plants against UV radiation. Consumption of considerable amounts of the green parts of buckwheat plants can also cause fagopyrism, photosensitisation with skin irritation and edema. However, the content of fagopyrin is lower than those of other antioxidative compounds, thus avoiding the negative effects of fagopyrin on human health. Emodin, with structural similarity to metabolites from the group of naphthodianthrones, isolated from Tartary buckwheat grain has been shown to bind well to the active sites of the RNA binding domain of the nucleocapsid phosphoprotein of Sars-CoV-2. Emodin was detected in Tartary buckwheat leaves and bran. Its bioactivity should be further investigated.

The breeding objectives of buckwheat include stable yields, superior seed quality, lodging resistance, determinate growth habit, easy dehulling, low shattering of seeds, flood resistance, rutin content, low allergenic protein content, good aroma, and pre-harvest sprouting resistance. Breeding buckwheat to obtain plants with large grain embryos is a promising method to obtain genotypes with high flavonoid and emodin content.
